# The caddisfly fauna (Insecta, Trichoptera) of the rivers of the Black Sea basin in Kosovo with distributional data for some rare species

**DOI:** 10.3897/zookeys.182.2485

**Published:** 2012-04-10

**Authors:** Halil Ibrahimi, Mladen Kučinić, Agim Gashi, Linda Grapci-Kotori

**Affiliations:** 1 Department of Biology, Faculty of Mathematical and Natural Sciences, University of Prishtina, “Mother Theresa” p.n. 10000 Prishtina, Kosovo; 2 Department of Biology, Faculty of Science, University of Zagreb, (Group for Systematic Zoology & Entomology), Rooseveltov trg 6, 10000 Zagreb, Croatia

**Keywords:** Trichoptera, aquatic insects, Kosovo, Balkan Peninsula, Europe

## Abstract

Adult caddisflies were collected from 12 stations in the Black Sea basin in Kosovo using UV light traps. Sixty-five of the seventy-six species reported in this paper are first records for the Kosovo caddisfly fauna. The unexpected discovery of several species during this investigation: *Agapetus delicatulus* McLachlan, 1884, *Psychomyia klapaleki* Malicky, 1995, *Tinodes janssensi* Jacquemart, 1957, *Hydropsyche emarginata* Navas, 1923, *Drusus botosaneanui* Kumanski, 1968, *Potamophylax rotundipennis* (Brauer, 1857), *Potamophylax schmidi* Marinković-Gospodnetić, 1970, *Ceraclea albimacula* (Rambur, 1842), *Helicopsyche bacescui* Orghidan & Botosaneanu, 1953, *Adicella filicornis* (Pictet, 1834), *Beraea maurus* (Curtis, 1834) and *Beraeamyia hrabei* Mayer, 1937 illustrates that collections from poorly investigated areas in Europe will almost certainly revise the existing knowledge on the distribution of these and other species.

## Introduction

To our knowledge, Europe (in the zoogeographical sense) currently holds more than 1100 caddisfly species (e.g. Malicky 2004; Oláh 2010; Wiberg-Larsen 2008). Within Europe, the Balkan Peninsula is a unique region that is known for its plant and animal species richness, due in part to its biogeographical and ecological features such as the presence of different regions with a variety of condition, complex geological history and interaction between populations, species and ecosystems (Savić 2008) . Historic faunistic data for several groups of aquatic insects, including caddisflies, in the Balkan Peninsula date back over a century (e.g. Klapálek 1899, 1902; Radovanović 1931, 1935, 1953). Only recently, however, have distributions and zoogeographic characteristics, descriptions of new taxa, and larval-adult associations been examined more thoroughly in the Balkans (e.g. Chvojka 1997; Ćuk and Vučković 2009; Graf et al. 2008; Kučinić et al. 2011a, 2011b; Kučinić and Malicky 2002; Malicky 2009; Oláh 2010; Previšić et al. 2007; Vučković et al. 2011; Waringer et al. 2009; Živić et al. 2009).

Poorly investigated areas in Europe still remain: until recently Albania and Kosovo were among the least studied areas in the Balkan Peninsula and Europe in general. Recent caddisfly investigations in Albania have documented many rare or unexpected species (e.g. Oláh 2010; Oláh et al. 2011). This suggests that caddisfly zoogeographic studies in poorly investigated areas of the Balkan Peninsula will continuously revise the present knowledge of the distributions of many European species. Kosovo still remains a poorly investigated area in Europe in regard to caddisfly fauna. Until recently, only 37 Trichoptera species were known from Kosovo (Ibrahimi et al. in press; Malicky 1986, 1999; Marinković-Gospodnetić 1975, 1980; Oláh 2010; Pongrácz 1923). This study is part of the first large-scale investigation of caddisfly fauna distribution in this part of the Balkan Peninsula based on adult specimen collections.

All rivers in Kosovo belong to the drainage basins of three seas: the Black Sea, the Adriatic Sea and the Aegean Sea. This study examined the caddisfly fauna of the Black Sea drainage basin, which is the largest in the area and covers about 50% of the Kosovo’s territory of 10,908 km^2^. Rivers that belong to the Black Sea drainage basin are the Ibri, Sitnica, Llapi, Drenica, and Morava e Binçës, as well as other smaller streams and tributaries.

## Material and methods

**Figure 1.**
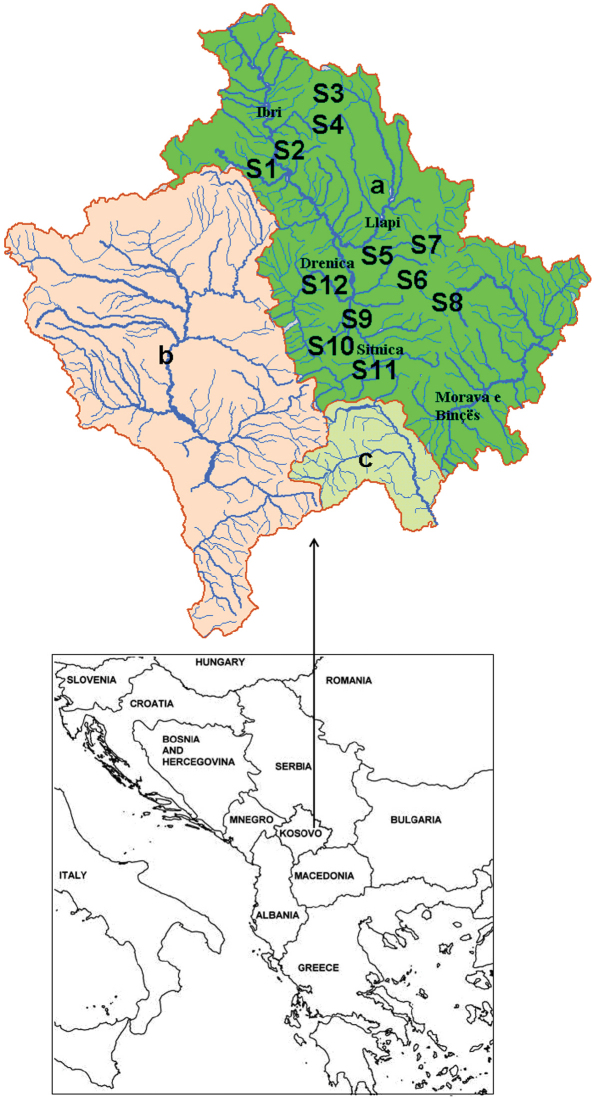
Sampling sites: S1 Koshtovë, S2 Mazhiq, S3 Murgull, S4 Kaqandoll, S5 Siqevë, S6 Orllan, S7 Llukar, S8 Marefc, S9 Blinajë, S10 Mollopolc, S11 Caralevë and S12 Grabofc. Drainage basins: a) Black Sea basin, b) Adriatic Sea basin and c) Aegean Sea basin.

Adult caddisflies were collected using UV light traps at 12 stations in the Black Sea drainage basin of Kosovo (Figure 1 and Table 1). The sampling was carried out between March 2009 and November 2010. Light traps were placed on stream banks and operated for approximately one hour and fifteen minutes after dusk. All samples were preserved in 80 % ethanol. The specimens were identified under a stereomicroscope with determination keys from Malicky (2004) and Kumanski (1985, 1988). Specimens were collected by Halil Ibrahimi and were determined by Halil Ibrahimi and Mladen Kučinić unless otherwise noted. The collection is deposited at the Laboratory of Zoology of the Faculty of Natural and Mathematical Sciences, University of Prishtina, Kosovo.

**Table 1.** Locality data for the 12 sampling stations.

**Code**	**Sampling Stations**	**River/Stream**	**Latitude °N, Longitude °E**		**Altitude m**
S1	Koshtovë	Ibër River	42.8688°N, 20.8271°E		859
S2	Mazhiq	Mazhiq Stream	42.9401°N, 20.9331°E		853
S3	Murgull	Murgull River	43.0743°N, 21.066°E		882
S4	Kaqandoll	Kaqandoll River	42.9666°N, 21.0702°E		1069
S5	Siqevë	Siqevë Stream	42.7369°N, 21.2343°E		798
S6	Orllan	Stream	42.7709°N, 21.3262°E		844
S7	Llukar	Prishtina River	42.6987°N, 21.2367°E		728
S8	Marefc	Marefc Stream	42.6321°N, 21.4257°E		691
S9	Blinajë	The first lake	42.5185°N, 20.9788°E		721
S10	Mollopolc	Mollopolc Stream	42.4056°N, 21.0408°E		655
S11	Caralevë	Caralevë Stream	42.4477°N, 20.9482°E		756
S12	Grabofc	Drenica River	42.6749°N, 20.9664°E		600

Systematics and nomenclature follows Malicky (2004) and Marinković – Gospodnetić (1970). Within the families the genera and taxa are given in alphabetical order. Biogeographic analysis and distributional data follow Botosaneanu and Malicky (1978), Cianficoni (2002), Graf et al. (2008), Krušnik (1987), Kumanski (1985, 1988), Kučinić (2002), Kučinić et al. (2008, 2011a), Malicky (2011), Marinković-Gospodnetić (1979), Oláh (2010), Previšić et al. (2007), Stanić-Koštroman (2009), Waringer et al. (2009) and Živić et al. (2006, 2009). Female specimens belonging to the genera where identification until species level is impossible according to Malicky (2004) were not counted in taxa list except the female specimen of *Potamophylax* sp., which is morphologically different from *Potamophylax luctuosus* (Piller & Mitterpacher, 1783), *Potamophylax pallidus* (Klapalek, 1899), *Potamophylax rotundipennis* (Brauer, 1857) and *Potamophylax schmidi* Marinković – Gospodetić, 1970, and consequently was counted as a twelfth taxa of Limnephilidae family.

## Results

A total of 8,869 specimens belonging to seventy-six species are reported. The distribution of species per family is as follows: Rhyacophilidae (8) Glossosomatidae (5), Hydroptilidae (1), Philopotamidae (5), Hydropsychidae (11), Polycentropodidae (4), Psychomyiidae (6), Phryganeidae (1), Brachycentridae (2), Limnephilidae (12), Goeridae (5), Lepidostomatidae (1), Leptoceridae (9), Sericostomatidae (2), Helicopsychidae (1) and Beraeidae (3) (Table 2).

Sixty-five out of the seventy-six total species reported here are new records for the Kosovo caddisfly fauna (Table 2). A singe female specimen of *Potamophylax* sp. (Limnephilidae) that does not match the descriptions for any described females in this genus was found in station S9. The female specimen is similar to *Potamophylax rotundipennis* in appearance,but the genital morphology clearly distinct (Malicky, per. com.). This specimen could potentially be new to science, but we refrain from a description until associated males are available. Eight species in the genus *Rhyacophila* presented in this paper were previously reported and discussed (Ibrahimi et al., in press; Marinković-Gospodnetić, 1975), but we provided detailed collection data together with data for specimens collected at new localities in 2009 and 2010.

**Table 2.** Systematic list of described caddisflies collected in the Black Sea Basin from 2009–2010. Species new to the fauna of Kosovo are indicated by an asterisk *.

Family Rhyacophilidae	
*Rhyacophila armeniaca* Guerin - Meneville, 1834	
	S3 Murgull: 20.VII.2009. 1 ♀, 4 ♂♂; S4 Kaqandoll: 20.VII.2009. 2 ♂♂; S5 Siqevë: 21.VI.2009. 1 ♂.
*Rhyacophila fasciata* Hagen, 1859	
	S1 Koshtovë: 15.III.2010. 10 ♂♂, 16.IV.2010. 5 ♀♀, 13 ♂♂, 13.V.2010. 6 ♀♀, 15 ♂♂, 18.VI.2010. 7 ♀♀, 11 ♂♂, 20.VII.2010. 1 ♀, 7 ♂♂, 16.VIII.2010. 3 ♀♀, 7 ♂♂, 17.IX.2010. 4 ♀♀, 16 ♂♂, 18.X.2010. 4 ♀♀, 4 ♂♂; S2 Mazhiq: 18.VI.2009. 6 ♀♀, 7 ♂♂, 20.VII.2009. 8 ♀♀, 3 ♂♂, 16.VIII.2009. 13 ♀♀, 21 ♂♂, 17.IX.2009. 14 ♀♀, 32 ♂♂; S3 Murgull: 20.VII.2009. 4 ♀♀, 8 ♂♂, 17.VIII.2009. 3 ♀♀, 9 ♂♂, 18.IX.2009. 2 ♀♀, 6 ♂♂, 19.X.2009. 8 ♀♀, 10 ♂♂, 19.XI.2009. 2 ♂♂; S4 Kaqandoll: 19.VI.2009. 1 ♀, 5 ♂♂, 20.VII.2009. 6 ♀♀, 9 ♂♂, 18.IX.2009. 21 ♀♀, 33 ♂♂; S5 Siqevë: 15.VIII.2009. 11 ♀♀, 23 ♂♂, 16.IX.2009. 13 ♀♀, 7 ♂♂, 17.X.2009. 5 ♀♀, 7 ♂♂; S6 Orllan: 21.VI.2009. 3 ♀♀, 6 ♂♂, 17.VII.2009. 2 ♀♀, 7 ♂♂, 15.VIII.2009. 13 ♀♀, 24 ♂♂, 16.IX.2009. 12 ♀♀, 23 ♂♂; S7 Llukar: 15.VIII.2009. 5 ♀♀, 9 ♂♂, 16.IX.2009. 11 ♀♀, 4 ♂♂; S8 Marefc: 09.IV.2010. 3 ♂♂, 09.V.2010. 6 ♂♂, 12.VI.2010. 3 ♂♂, 11.VII.2010. 1 ♀ ,9 ♂♂, 10.VIII.2010. 12 ♂♂, 09.IX.2010. 29 ♀♀, 28 ♂♂; S10 Mollopolc: 13.IV.2010. 5 ♂♂, 16.V.2010. 4 ♀♀, 31 ♂♂, 11.VI.2010. 7 ♂♂, 14.VII.2010. 4 ♂♂, 15.VIII.2010. 3 ♂♂, 14.IX.2010. 4 ♀♀, 5 ♂♂; S11 Caralevë: 16.V.2010. 7 ♀♀, 9 ♂♂, 11.VI.2010. 5 ♀♀, 9 ♂♂, 14.VII.2010. 13 ♀♀, 8 ♂♂, 15.VIII.2010. 4 ♂♂, 14.IX.2010. 8 ♀♀, 4 ♂♂; S12 Grabofc: 06.VI.2009. 3 ♀♀, 6 ♂♂, 03.VIII.2009. 13 ♀♀, 9 ♂♂.
*Rhyacophila fischeri* Botosaneanu, 1957	
	S2 Mazhiq: 13.V.2009. 2 ♀♀, 16 ♂♂; S3 Murgull: 14.V.2009. 3 ♀♀, 20 ♂♂, 19.V.2009. 2 ♀♀, 34 ♂♂; S4 Kaqandoll: 14.V.2009. 4 ♀♀, 9 ♂♂; S5 Siqevë: 13.V.2009. 2 ♀♀, 7 ♂♂; S7 Llukar: 17.07.2009. 14 ♀♀, 26 ♂♂.
*Rhyacophila laevis* Pictet, 1834	
	S5 Siqevë: 13.V.2009. 4 ♀♀, 6 ♂♂; S8 Marefc: 09.V.2010. 8 ♀♀, 9 ♂♂.
*Rhyacophila loxias* Schmid, 1970	
	S2 Mazhiq: 18.VI.2009. 4 ♂♂; S3 Murgull: 19.VI.2009. 1 ♂.
*Rhyacophila nubila* Zetterstedt, 1840	
	S1 Koshtovë: 16.IV.2010. 2 ♀♀, 6 ♂♂, 13.V.2010. 11 ♀♀, 32 ♂♂, 18.VI.2010. 8 ♀♀, 16 ♂♂, 20.VII.2010. 6 ♀♀, 21 ♂♂; S3 Murgull: 18.IX.2009. 5 ♀♀, 12 ♂♂.
*Rhyacophila polonica* McLachlan 1879	
	S2 Mazhiq: 18.VI.2009. 5 ♀♀, 14 ♂♂, 20.VII.2009. 4 ♀♀, 22 ♂♂, 16.VIII.2009. 13 ♀♀, 4 ♂♂; S6 Orllan: 21.VI.2009. 12 ♀♀, 23 ♂♂, 17.VII.2009. 11 ♀♀, 30 ♂♂, 15.VIII.2009. 14 ♀♀, 2 ♂♂; S8 Marefc: 12.VI.2010. 4 ♂♂; S10 Mollopolc: 21.VI.2010. 1 ♀, 5 ♂♂.
*Rhyacophila tristis* Pictet, 1834	
	S2 Mazhiq: 15.V.2009. 3 ♀♀, 9 ♂♂; S3 Murgull: 14.V.2009. 12 ♀♀, 22 ♂♂, 19.VI.2009. 3 ♂♂, 20.VII.2009. 2 ♂♂; S4 Kaqandoll: 14.V.2009. 14 ♀♀, 23 ♂♂, 19.VI.2009. 5 ♀♀, 19 ♂♂; S5 Siqevë: 13.VI.2009. 9 ♀♀, 21 ♂♂, 21.VI.2009. 21 ♀♀, 42 ♂♂, 17.VII.2009. 3 ♀♀, 7 ♂♂; S7 Llukar: 13.V.2009. 5 ♀♀, 24 ♂♂; S10 Mollopolc: 18 ♀♀, 39 ♂♂, 11.VI.2009. 48 ♀♀, 53 ♂♂, 14.VII.2009. 15 ♀♀, 6 ♂♂, 15.VIII.2009. 2 ♂♂; S11 Caralevë: 14.V.2010. 5 ♀♀, 42 ♂♂.
Family Glossosomatidae	
*Agapetus delicatulus* McLachlan, 1884 *	
	S5 Siqevë: 21.VI.2009. 5 ♀♀ (2 ♀♀ det. H. Malicky), 12.VII.2009. 1 ♂.
*Agapetus ochripes* Curtis, 1834 *	
	S4 Kaqandoll: 19.VI.2009. 2 ♂♂.
*Glossosoma conformis* Neboiss, 1963 *	
	S4 Kaqandoll: 19.VI.2009. 2 ♂♂.
*Synagapetus iridipennis* McLachlan, 1879 *	
	S2 Mazhiq: 18.VI.2009. 5 ♀♀, 13 ♂♂; S5 Siqevë: 21.VI.2009. 3 ♀♀, 18 ♂♂, 17.VII.2009. 2 ♀♀, 18 ♂♂; S6 Orllan: 21.VI.2009. 4 ♀♀, 14 ♂♂, 17.VII.2009. 5 ♂♂; S7 Llukar: 21.VI.2009. 32 ♀♀, 28 ♂♂; S10 Mollopolc: 11.VI.2010. 5 ♂♂ (2 ♂♂ det. H. Malicky); S12 Grabofc: 05.IV.2009. 7 ♀♀, 3 ♂♂, 06.V.2009. 4 ♀♀, 7 ♂♂, 06.VI.2009. 6 ♀♀, 13 ♂♂, 08.VII.2009. 5 ♀♀, 7 ♂♂.
*Synagapetus slavorum* Botosaneanu, 1960 *	
	S10 Mollopolc: 11.VI.2010. 2 ♂♂, 15.VIII.2010. 4 ♂♂.
Family Hydroptilidae	
*Hydroptila forcipata* (Eaton, 1873) *	
	S1 Koshtovë: 18.VI.2010. 55 ♀♀, 20.VII.2010. 49 ♀♀, 2 ♂♂, 16.VIII.2010. 76 ♀♀, 1 ♂, 17.IX.2010. 87 ♀♀, 2 ♂♂.
Family Philopotamidae	
*Philopotamus montanus* (Donovan, 1813) *	
	S2 Mazhiq: 15.III.2009. 6 ♂♂, 16.IV.2009. 6 ♂♂, 13.V.2009. 16 ♀♀, 11 ♂♂, 18.VI.2009. 13 ♀♀, 12 ♂♂, 20.VII.2009. 5 ♀♀, 4 ♂♂, 16.VIII.2009. 21 ♀♀, 22 ♂♂; S3 Murgull: 16.III.2009. 3 ♂♂, 17.IV.2009. 1 ♀, 9 ♂♂, 14.V.2009. 8 ♀♀, 31 ♂♂, 19.VI.2009. 1 ♀, 14 ♂♂, 20.VII.2009. 7 ♀♀, 3 ♂♂; S4 Kaqandoll: 16.III.2009. 5 ♂♂, 17.IV.2009. 20 ♂♂, 14.V.2009. 6 ♀♀, 18 ♂♂, 19.VI.2009. 11 ♀♀, 13 ♂♂; S5 Siqevë: 14.III.2009. 4 ♂♂, 16.IV.2009. 2 ♀♀, 18 ♂♂, 13.V.2009. 21 ♀♀, 13 ♂♂, 21.VI.2009. 13 ♀♀, 7 ♂♂, 17.VII.2009. 8 ♀♀, 11 ♂♂; S6 Orllan: 13.V.2009. 12 ♂♂, 21.VI.2009. 13 ♀♀, 6 ♂♂, 17.VII.2009. 2 ♀♀, 5 ♂♂; S7 Llukar: 16.IV.2009. 21 ♂♂, 13.V.2009. 32 ♀♀, 14 ♂♂; S10 Mollopolc: 12.III.2010. 6 ♂♂, 13.IV.2010. 5 ♂♂, 16.V.2010. 24 ♀♀, 19 ♂♂, 11.VI.2010. 9 ♀♀, 8 ♂♂, 14.VII.2010. 3 ♂♂, 15.VIII.2010. 2 ♀♀, 8 ♂♂, 14.IX.2010. 4 ♂♂; S12 Grabofc: 06.06.2009. 4 ♀♀, 3 ♂♂, 08.VII.2009. 6 ♀♀, 4 ♂♂.
*Philopotamus variegatus* (Scopoli, 1763) *	
	S8 Marefc: 09.V.2010. 2 ♂♂.
*Wormaldia occipitalis* (Pictet, 1834) *	
	S3 Murgull: 19.VI.2009. 11 ♂♂, 20.VII.2009. 15 ♀♀, 3 ♂♂; S5 Siqevë: 17.VII.2009. 5 ♂♂, 17.X.2009. 6 ♀♀, 7 ♂♂; S6 Orllan: 16.IX.2009. 5 ♀♀, 16 ♂♂; S7 Llukar: 17.VII.2009. 15 ♀♀, 21 ♂♂, 15.VIII.2009. 6 ♀♀, 9 ♂♂; S8 Marefc: 11.VII.2010. 4 ♂♂, S9 Blinajë: 10.V.2010. 12 ♀♀, 18 ♂♂, 13.VI.2010. 21 ♀♀, 34 ♂♂, 11.VIII.2010. 4 ♀♀ , 14 ♂♂,10.IX.2010. 21 ♀♀, 34 ♂♂, S10 Mollopolc: 14.VII.2010. 7 ♂♂, 15.VIII.2010. 21 ♀♀, 14 ♂♂, 14.IX.2010. 32 ♀♀, 6 ♂♂; S11 Caralevë: 11.VI.2010. 7 ♂♂; S12 Grabofc; 08.VII.2009. 4 ♀♀, 2 ♂♂.
*Wormaldia pulla* (McLachlan, 1878) *	
	S4 Kaqandoll: 19.VI.2009. 1 ♂.
*Wormaldia subnigra* McLachlan, 1865 *	
	S6 Orllan: 17.VII.2009. 5 ♂♂.
Family Hydropsychidae	
*Cheumatopsyche lepida* (Pictet, 1834) *	
	S6 Orllan: 21.VI.2009. 12 ♀♀, 5 ♂♂, 17.VII.2009. 5 ♀♀, 13 ♂♂, 15.VIII.2009. 9 ♀♀; S7 Llukar: 13.V.2009. 4 ♀♀, 7 ♂♂, 21.VI.2009. 21 ♀♀, 32 ♂♂; S8 Marefc: 09.V.2010. 170 ♀♀, 38 ♂♂, 12.VI.2010. 98 ♀♀, 23 ♂♂, 11.VII.2010. 43 ♀♀, 21 ♂♂, 10.VIII.2010. 56 ♀♀, 12 ♂♂, 09.IX.2010. 32 ♀♀, 6 ♂♂.
*Diplectrona atra* McLachlan, 1878 *	
	S3 Murgull: 19.VI.2009. 14 ♀♀, 11 ♂♂, 20.VII.2009. 1 ♀, 4 ♂♂, 17.VIII.2009. 14 ♀♀; S8 Marefc: 09.V.2010. 23 ♀♀, 8 ♂♂, 12.VI.2010. 12 ♀♀, 2 ♂♂, 11.VII.2010. 16 ♀♀, 2 ♂♂.
*Hydropsyche angustipennis* (Curtis, 1834) *	
	S9 Blinajë: 13.VII.2010. 18 ♂♂ (1 ♂ det. H. Malicky), 11.VIII.2010. 12 ♂♂.
*Hydropsyche bulbifera* McLachlan, 1878 *	
	S5 Siqevë: 16.IX.2009. 6 ♂♂; S6 Orllan: 21.VI.2009. 1 ♂, 15.VIII.2009. 5 ♂♂; S12 Grabofc: 05.IV.2009. 6 ♂♂, 03.VIII.2009. 4 ♂♂.
*Hydropsyche emarginata* Navas, 1923 *	
	S7 Llukar: 21.VI.2009. 3 ♂♂; S8 Marefc: 09.V.2010. 11 ♂♂, 12.VI.2010. 23 ♂♂, 11.VII.2010. 12 ♂♂, 10.VIII.2010. 5 ♂♂, 09.IX.2010. 5 ♂♂; S12 Grabofc: 06.VI.2009. 9 ♂♂.
*Hydropsyche fulvipes* Curtis, 1834 *	
	S7 Llukar: 21.VI.2009. 1 ♂ (det. H. Malicky).
*Hydropsyche incognita* Pitsch, 1993 *	
	S8 Marefc: 12.VI.2010. 4 ♂♂.
*Hydropsyche instabilis* (Curtis, 1834)	
	S4 Kaqandoll: 17.VIII.2009. 5 ♂♂.
*Hydropsyche peristerica* Botosaneanu & Marinković-Gospodnetić, 1968 *	
	S3 Murgull: 14.V.2009. 1 ♂, 20.VII.2009. 3 ♂♂; S5 Siqevë: 13.V.2009. 1 ♂ (det. H. Malicky).
*Hydropsyche saxonica* McLachlan, 1884 *	
	S4 Kaqandoll: 17.VIII.2009. 3 ♂♂, S6 Orllan: 21.VI.2009. 5 ♂♂, 15.VIII.2009. 5 ♂♂; S9 Blinajë: 13.VII.2010. 14 ♂♂ (1 ♂ det. H. Malicky).
*Hydropsyche tabacarui* Botosaneanu, 1960 *	
	S3 Murgull: 14.V.2009. 6 ♂♂; S10 Mollopolc: 11.VI.2010. 7 ♂♂.
*Hydropsyche* sp. ♀	
	S1 Koshtovë: 13.V.2010. 3 ♀♀; S2 Mazhiq: 18.VI.2009. 10 ♀♀, 20.VII.2009. 21 ♀♀, 16.VIII.2009. 12 ♀♀; S3 Murgull: 19.VI.2009. 8 ♀♀, 20.VII.2009. 8 ♀♀, 17.VIII.2009. 12 ♀♀; S4 Kaqandoll: 19.VI.2009. 17 ♀♀, 17.VIII.2009. 32 ♀♀; S5 Siqevë: 16.IX.2009. 13 ♀♀; S6 Orllan: 17.VII.2009. 13 ♀♀, 15.08.2009. 24 ♀♀; S7 Llukar: 21.VI.2009.32 ♀♀, 17.VII.2009. 14 ♀♀; S8 Marefc: 09.V.2010. 12 ♀♀, 12.VI.2010. 5 ♀♀, 11.VII.2010. 22 ♀♀, 10.VIII.2010. 5 ♀♀, 09.IX.2010. 8 ♀♀; S9 Blinajë: 13.VI.2010. 11 ♀♀, 12.VII.2010. 14 ♀♀, 11.VIII.2010. 21 ♀♀; S10 Mollopolc: 16.V.2010. 3 ♀♀, 11.VI.2010. 5 ♀♀, 14.VII.2010. 8 ♀♀; S11 Caralevë: 16.V.2010. 12 ♀♀, 11.VI.2010. 23 ♀♀, 14.VII.2010. 43 ♀♀, S12 Grabofc: 06.V.2009. 21 ♀♀, 06.VI.2009, 23 ♀♀, 08.VII.2009. 32 ♀♀, 03.VIII.3009. 13 ♀♀.
Family Polycentropodidae	
*Cyrnus trimaculatus* (Curtis, 1834) *	
	S9 Blinajë: 10.V.2010. 12 ♀♀, 19 ♂♂, 13.VI.2010. 21 ♀♀, 13 ♂♂. 12.VII.2010. 14 ♀♀, 18 ♂♂, 11.VIII.2010. 12 ♀♀, 5 ♂♂, 10.IX.2010. 7 ♀♀, 21 ♂♂; S12 Grabofc: 05.IV.2009. 3 ♀♀, 5 ♂♂, 06.V.2009. 4 ♀♀, 7 ♂♂, 06.VI.2009. 2 ♀♀, 4 ♂♂, 08.VII.2009. 21 ♀♀, 26 ♂♂, 03.VIII.3009. 5 ♀♀, 9 ♂♂.
*Polycentropus excisus* Klapalek, 1894 *	
	S10 Mollopolc: 13.X.2010. 1 ♂ (det. H. Malicky).
*Polycentropus flavomaculatus* (Pictet, 1834 ) *	
	S9 Blinajë: 12.VII.2010. 7 ♀♀, 10 ♂♂.
*Plectrocnemia conspersa* (Curtis, 1834) *	
	S2 Mazhiq: 20.VII.2009. 8 ♂♂, S8 Marefc: 11.VII.2010. 3 ♂♂.
Family Psychomyiidae	
*Lype reducta* (Hagen, 1868) *	
	S2 Mazhiq: 18.VI.2009. 12 ♀♀, 17 ♂♂, 20.VII.2009. 5 ♀♀, 11 ♂♂; S3 Murgull: 19.VI.2009. 1 ♂; S7 Llukar: 17.VII.2009 5 ♂♂ (1 ♂ det. H. Malicky); S9 Blinajë: 11.VIII.2010. 18 ♀♀, 13 ♂♂; S12 Grabofc: 08.VII.2009. 4 ♀♀, 7 ♂♂.
*Psychomyia klapaleki* Malicky, 1995 *	
	S1 Koshtovë: 20.VI.2010. 21 ♀♀, 2 ♂♂ (2 ♂♂ + 4 ♀♀ det. H. Malicky), 16.VIII.2010. 44 ♀♀, 2 ♂♂, 17.IX.2010. 54 ♀♀, 2 ♂♂.
*Psychomyia pusilla* (Fabricius, 1781) *	
	S6 Orllan: 21.VI.2009. 31 ♀♀, 5 ♂♂, 15.VIII.2009. 24 ♀♀, 2 ♂♂, 16.IX.2009. 44 ♀♀; 17.X.2009. 23 ♀♀; S7 Llukar: 21.VI.2009. 12 ♀♀, 17.VII.2009. 21 ♀♀, 1 ♂, 15.VIII.2009. 23 ♀♀, 1 ♂; S8 Marefc: 09.V.2010. 154 ♀♀, 3 ♂♂, 12.VI.2010. 342 ♀♀, 6 ♂♂, 11.VII.2010. 238 ♀♀, 8 ♂♂, 10.VIII.2010. 213 ♀♀, 5 ♂♂, 09.IX.2010. 459 ♀♀, 6 ♂♂; S12 Grabofc: 08.VII.2009. 32 ♀♀, 5 ♂♂, 03.VIII.3009. 76 ♀♀, 4 ♂♂.
*Tinodes janssensi* Jacquemart, 1957 *	
	S9 Blinajë: 10.V.2010. 5 ♀♀, 2 ♂♂, 13.VI.2010. 2 ♀♀.
*Tinodes rostocki* McLachlan, 1878 *	
	S2 Mazhiq: 13.V.2009. 3 ♀♀, 13 ♂♂; S3 Murgull: 14.V.2009. 9 ♀♀, 6 ♂♂, 20.VII.2009. 5 ♀♀, 3 ♂♂; S4 Kaqandoll: 14.V.2009. 14 ♀♀, 7 ♂♂; S5 Siqevë: 13.V.2009. 3 ♀♀, 8 ♂♂, 21.VI.2009. 12 ♀♀, 31 ♂♂, 17.VII.2009. 3 ♀♀, 4 ♂♂; S6 Orllan: 21.VI.2009. 2 ♂♂, 17.VII.2009. 4 ♂♂; S10 Mollopolc: 4 ♂♂, 14.IX.2009. 5 ♀♀.
*Tinodes unicolor* (Pictet, 1834) *	
	S10 Mollopolc: 13.X.2010. 6 ♀♀, 7 ♂♂.
*Tinodes* sp.♀	
	P11 Caralevë: 12.VI.2010. 8 ♀♀, 12.VII.2010. 7 ♀♀, 14.VIII.2010. 8 ♀♀.
Family Phryganeidae	
*Agrypnia varia* (Fabricius, 1793) *	
	S1 Koshtovë: 16.VIII.2010. 8 ♀♀, 12 ♂♂.
Family Brachycentridae	
*Micrasema minimum* McLachlan, 1876 *	
	S7 Llukar: 21.VI.2009. 13 ♀♀, 27 ♂♂, 17.VII.2009. 4 ♀♀, 7 ♂♂.
*Micrasema sericeum* Klapalek, 1902 *	
	S1 Koshtovë: 18.VI.2009. 13 ♀♀, 33 ♂♂, 20.VII.2010. 12 ♀♀, 23 ♂♂, 13 ♀♀, 33 ♂♂, 20.VII.2010. 12 ♀♀, 23 ♂♂.
Family Limnephilidae	
*Anabolia furcata* Brauer, 1857 *	
	S12 Grabofc: 06.VI.2010. 6 ♀♀, 03.VIII.2009. 5 ♀♀, 9 ♂♂, 06.IX.2009. 13 ♀♀, 18 ♂♂.
*Chaetopteryx bosniaca* Marinković, 1955 *	
	S3 Murgull: 19.X.2009. 1 ♀, 11.X.2010. 11 ♀♀, 12 ♂♂; S9 Blinajë: 11.X.2010. 11 ♀♀, 12 ♂♂.
*Drusus botosaneanui* Kumanski, 1968 *	
	S2 Mazhiq: 16.VIII.2009. 12 ♀♀, 4 ♂♂, 17.IX.2009. 23 ♀♀, 18 ♂♂; S3 Murgull: 17.VIII.2009. 5 ♂♂, 18.IX.2009. 2 ♀♀, 9 ♂♂, 19.IX.2009. 14 ♀♀, 7 ♂♂; S4 Kaqandoll: 18.IX.2009. 14 ♀♀, 8 ♂♂, 19.X.2009. 2 ♂♂, 20.XI.2009. 4 ♀♀; S5 Siqevë: 15.VIII.2009. 13 ♀♀, 7 ♂♂, 16.IX.2009. 20 ♀♀, 15 ♂♂, 18.XI.2009. 1 ♀; S6 Orllan: 15.VIII.2009. 12 ♀♀, 5 ♂♂, 16.IX.2009. 23 ♀♀, 18 ♂♂; S7 Llukar: 15.VIII.2009. 16 ♀♀, 3 ♂♂, 16.IX.2009. 12 ♀♀, 7 ♂♂; S10 Mollopolc: 14.IX.2010. 13 ♀♀, 7 ♂.
*Glyphotaelius pellucidus* (Retzius, 1783) *	
	S12 Grabofc: 06.VI.2009. 4 ♀♀, 3 ♂♂.
*Grammotaulius nigropuncatus* (Retzius, 1873) *	
	S10 Mollopolc: 13.X.2010. 1 ♀; 06.V.2009. 3 ♂♂.
*Halesus digitatus* (Schrank, 1781) *	
	S2 Mazhiq: 17.IX.2009. 1 ♀; S5 Siqevë: 17.X.2009. 1 ♀; S7 Llukar: 17.X.2009. 4 ♀♀, 18.XI.2009. 4 ♀♀.
*Limnephilus vittatus* (Fabricius, 1798) *	
	S9 Blinajë: 10.V.2010. 3 ♂♂.
*Potamophylax luctuosus* (Piller & Mitterpacher, 1783) *	
	S10 Mollopolc: 13.VI.2010. 1 ♂ (det. H. Malicky).
*Potamophylax pallidus* (Klapalek, 1899)	
	S11 Caralevë: 14.XI.2010. 1 ♀.
*Potamophylax rotundipennis* (Brauer, 1857) *	
	S5 Siqevë: 15.IX.2009. 2 ♀♀ (det. H. Malicky); S8 Marefc: 08.X.2010. 1 ♂.
*Potamophylax schmidi* Marinković-Gospodnetić, 1970 *	
	S5 Siqevë: 13.VI.2009. 1 ♀ (det. H. Malicky).
*Potamophylax* sp. *	
	S9 Blinajë: 12.XI.2010. 1 ♀ (det. H. Malicky).
Family Goeridae	
*Goera pilosa* (Fabricius, 1775) *	
	S6 Orllan: 21.VI.2009. 5 ♀♀, 7 ♂♂; S7 Llukar: 21.VI.2009. 2 ♀♀, 7 ♂♂; S12 Grabofc: 08.VII.2009. 12 ♀♀, 21 ♂♂, 03.VIII.2009. 13 ♀♀, 7 ♂♂.
*Lithax obscurus* (Hagen, 1859) *	
	S6 Orllan: 21.VI.2009. 3 ♂♂; S7 Llukar: 13.V.2009. 37 ♀♀, 36 ♂♂.
*Silo graellsi* Pictet, 1865 *	
	S2 Mazhiq: 18.VI.2009. 8 ♀♀, 14 ♂♂; S4 Kaqandoll: 14.V.2009. 1 ♂; S5 Siqevë: 21.VI.2009. 6 ♀♀, 7 ♂♂.
*Silo pallipes* (Fabricius, 1781) *	
	S3 Murgull: 14.V.2009. 2 ♀♀, 6 ♂♂.
*Silo piceus* (Brauer, 1857) *	
	S1 Koshtovë: 16.IV.2010. 6 ♀♀, 2 ♂♂, 13.V.2010. 18 ♀♀, 11 ♂♂, 18.VI.2010. 4 ♀♀, 1 ♂; S4 Kaqandoll: 19.VI.2009. 44 ♀♀, 21 ♂♂; S6 Orllan: 19.VI.2009: 4 ♀♀, 23 ♂♂; S7 Llukar: 1 ♀, 14 ♂♂, 21.VI.2009. 4 ♀♀, 7 ♂♂, 17.VII.2009. 12 ♀♀, 24 ♂♂, 15.VIII.2009. 3 ♀♀, 5 ♂♂; S12 Grabofc: 03.VIII.2009. 31 ♀♀, 19 ♂♂.
Family Lepidostomatidae	
*Lepidostoma basale* (Kolenati, 1848)	
	S3 Murgull: 20.VII.2009. 8 ♀♀, 18.IX.2009. 1 ♂; S4 Kaqandoll: 14.V.2009. 24 ♀♀, 29 ♂♂, 19.VI.2009. 6 ♀♀, 1 ♂, 17.VIII.2009. 3 ♀♀, 4 ♂♂.
Family Leptoceridae	
*Adicella filicornis* (Pictet, 1834) *	
	S10 Mollopolc: 13.X.2010. 2 ♂♂ (det. H. Malicky).
*Adicella syriaca* Ulmer, 1907 *	
	S12 Grabofc: 06.VI.2009. 6 ♀♀, 4 ♂♂.
*Athripsodes bilineatus* (Linnaeus, 1758) *	
	S6 Orllan: 21.VI.2009. 9 ♀♀, 26 ♂♂ (6 ♂♂, 1 ♀ det. H. Malicky); 17.07.2009. 13 ♀♀, 19 ♂♂; S7 Llukar: 21.VI.2009. 5 ♀♀, 2 ♂♂, 17.VII.2009. 14 ♀♀, 8 ♂♂; S9 Blinajë: 13.VI.2010. 3 ♀♀, 13 ♂♂; S10 Mollopolc: 14.VII.2010. 8 ♀♀, 21 ♂♂, 15.VIII.2010. 6 ♀♀, 9 ♂♂, 13.X.2010. 3 ♀♀, 1 ♂, 13.XI.2010. 4 ♂♂; S12 Grabofc: 06.VI.2009. 13 ♀♀, 17 ♂♂; 08.VII.2009. 6 ♂♂.
*Athripsodes cinereus* (Curtis, 1834) *	
	S8 Marefc: 09.V.2010. 5 ♂♂, 12.VI.2010. 4 ♀♀, 8 ♂♂, 11.VII.2010. 2 ♀♀, 8 ♂♂, 10.VIII.2010. 12 ♀♀, 24 ♂♂.
*Ceraclea albimacula* (Rambur, 1842) *	
	S12 Grabofc: 06.VI.2009. 2 ♀♀, 7 ♂♂, 08.VII.2009. 4 ♀♀, 3 ♂♂, 03.VIII.2009. 1 ♂.
*Ceraclea dissimilis* (Stephens, 1836) *	
	S12 Grabofc: 03.VIII.3009. 1 ♂.
*Leptocerus interruptus* (Fabricius, 1775) *	
	S3 Murgull: 20.VII.2009. 2 ♂♂; S8 Marefc: 11.VII.2010. 7 ♂♂; S12 Grabofc: 06.VI.2009. 13 ♀♀, 21 ♂♂, 08.VII.2009. 13 ♀, 27 ♂♂.
*Mystacides azurea* (Linnaeus, 1761) *	
	S6 Orllan: 17.VII.2009. 6 ♀♀, 29 ♂♂; S8 Marefc: 09.V.2010. 2 ♀♀, 5 ♂♂, 12.VI.2010. 5 ♀♀, 3 ♂♂, 11.VII.2010. 7 ♀♀, 3 ♂♂, 10.VIII.2010. 4 ♀♀, 9 ♂♂; S9 Blinajë: 10.V.2010. 21 ♀♀, 43 ♂♂, 13.VI.2010. 13 ♀♀, 29 ♂♂, 12.VII.2010. 6 ♀♀, 8 ♂♂, 11.VIII.2010. 12 ♀♀, 23 ♂♂, 10.IX.2010. 4 ♀♀, 9 ♂♂; S12 Grabofc: 03.VIII.2009. 5 ♀♀, 8 ♂♂, 06.IX.2009. 5 ♀♀, 8 ♂♂.
*Mystacides nigra* (Linnaeus, 1758) *	
	S9 Blinajë: 10.V.2010. 11 ♀♀, 32 ♂♂, 12.VII.2010. 3 ♀♀, 9 ♂♂; S12 Grabofc: 06.V.2009. 6 ♀♀, 06.VI.2009. 4 ♀♀, 3 ♂♂, 08.VII.2009. 5 ♀♀, 9 ♂♂.
Family Sericostomatidae	
*Oecismus monedula* (Hagen, 1859) *	
	S2 Mazhiq: 18.VI.2009. 3 ♂♂; S8 Marefc: 6 ♀♀, 9 ♂♂; S12 Grabofc: 05.IV.2009. 3 ♀♀, 6 ♂♂.
*Sericostoma flavicorne* Schneider, 1845 *	
	S1 Koshtovë: 13.V.2010. 3 ♀♀, 1 ♂, 18.VI.2010. 5 ♀♀, 6 ♂♂; S3 Murgull: 20.VII.2009. 3 ♀♀, 7 ♂♂; S4 Kaqandoll: 14.V.2009. 2 ♂♂, 19.VI.2009. 7 ♀♀, 12 ♂♂.
Family Helicopsychidae	
*Helicopsyche bacescui* Orghidan & Botosaneanu, 1953 *	
	S9 Blinajë: 10.V.2010. 4 ♂♂.
Family Beraeidae	
*Beraea maurus* (Curtis, 1834) *	
	S6 Orllan: 17.VII.2009. 2 ♂♂.
*Beraeamyia hrabei* Mayer, 1937 *	
	S7 Llukar: 17.VII.2009. 2 ♂♂ (1 ♂ det. H. Malicky); S10 Mollopolc: 11.VI.2010. 2 ♂♂, 14.VII.2010. 9 ♀♀, 6 ♂♂.
*Ernodes articularis* (Pictet, 1834) *	
	S5 Siqevë: 21.VI.2009. 2 ♂♂, 17.VII.2009. 1 ♂; S10 Mollopolc: 11.VI.2010. 5 ♂♂.

## Discussion

Most of the species collected during this investigation belong to the European fauna (27) followed by the Euro-Asian group with 23 species, the Balkanic group with 9 species, the Western-Palearctic group with 8 species, the Carpathian-Balkanic group with 5 species and the Palearctic group with 4 species.

Besides the common and widespread species that are also known from the surrounding countries in the region, we found several species that were previously considered endemics for certain regions on the Balkan Peninsula and/or South-Eastern Europe: *Tinodes janssensi*, *Psychomyia klapaleki*, *Potamophylax schmidi*, *Hydropsyche emarginata* and *Drusus botosaneanui*. We also found species whose occurrences were unexpected in this part of Europe: *Agapetus delicatulus*, *Potamophylax rotundipennis*, *Ceraclea albimacula*, *Helicopsyche bacescui*, *Adicella filicornis*, *Beraea maurus* and *Beraeamyia hrabei*.

For example, *Agapetus delicatulus* is a widespread species in Europe and its range extends to the Western Asia Minor. According to present knowledge it is, however, absent from most part of the Balkan Peninsula. The closest areas to Kosovo from which it is reported are Albania and Bulgaria (Chvojka 1997; Kumanski 1985). The limited number of localities and our finding this species in Kosovo suggests that its distribution is greater than previously thought and extends farther south on the Balkan Peninsula. The species *Psychomyia klapaleki* was described from Slovenia (Malicky 1995) but is also known from Croatia, Bosnia and Hercegovina and Serbia (Stanić-Koštroman 2009; Vučković 2011; Živić et al. 2006). The finding in Kosovo suggests that the distribution area of this species is wider than previously thought. Currently, Kosovo presents the southernmost point of its known distribution. *Tinodes janssensi* is a very rare Balkanic endemic species present in several localities in Greece (Malicky 2005), from where it was originally described, and is also reported from single localities in Albania (Chvojka 1997) and Bulgaria (Kumanski 1985). The finding in Kosovo represents the northernmost point of its known distribution. The species *Potamophylax schmidi* was described from Bosnia and Hercegovina (Marinković-Gospodnetić 1970). Besides this, *Potamophylax schmidi* is only known from a single locality in Croatia (Malicky, per. com.) not far away from the type locality. There are several local endemic species of the genus *Potamophylax* in the Balkan Peninsula that were thought to be strictly limited to the mountains or other areas where they were found previously (Kumanski and Malicky 1999). Because of this and because of the large intervening distance, this species was not expected in Kosovo. As a result of our finding, the distributional area of *Potamophylax schmidi* is considerably enlarged. This suggests that other endemic species of the genus *Potamophylax* in the Balkan Peninsula may have wider area of distribution than currently known. The species *Potamophylax rotundipennis* was also not expected in Kosovo. This species is widespread in North-Western Europe stretching towards the East as well. Despite very detailed studies in many parts of South-Eastern Europe, the species was previously not found beyond Croatia or Romania. The finding of *Potamophylax rotundipennis* in Kosovo is a first record for ecoregion 6 and represents the southernmost locality of its distribution, remarkably enlarging its distribution range. A single specimen of this species collected in Serbia also was found in a museum collection (Živić et al. 2006), suggesting that the distribution of *Potamophylax rotundipennis* is wider than previously thought.

The species *Drusus botosaneanui* was known previously from Greece, Bulgaria, Serbia and the Sultan Mountains in Turkey (Kumanski 1988; Malicky 2005; Sipahiler 2003), and seems to be widely distributed in the Balkan Peninsula. During this investigation it was found at seven stations, making it one of the most widely distributed species in Kosovo. Recently this species was found in Albania (Oláh 2010) and Macedonia (Halil Ibrahimi unpublished results). *Ceraclea albimacula* in Europe is present only in the north-western part of the continent. The only record for this species in South-Eastern Europe is from Bosnia and Hercegovina (Stanić-Koštroman 2009). The find in Kosovo suggests that there are isolated populations of this species towards the south-eastern part of the continent, well beyond its previously known distribution area. Two species of Beraeaidae: *Beraea maurus* and *Beraeamyia hrabei* according to present knowledge are almost completely lacking from most of the Balkan Peninsula, and finding these species in Kosovo enlarges remarkably their distribution. The species *Helicopsyche bacescui* in Europe has a disjunct distribution, and recent investigations in neighboring Serbia documented its wide distribution (Živić et al. 2009). Discovering this species in Kosovo considerably enlarges its distribution towards the south-west. The presence of *Hydropsyche emarginata* and *Adicella filicornis* in Kosovo also enlarges their known distributions in South-Eastern Europe.

This study, along with other investigations (e.g. Krušnik 1987; Krušnik and Urbanič 2002; Kumanski 1985, 1988; Kumanski and Malicky 1999; Malicky 2005; Marinković-Gospodnetić 1966, 1975, 1980; Obr 1969; Oláh 2010; Oláh et al. 2011; Stanić-Koštroman 2009; Živić et al. 2002, 2006, 2009) have greatly expanded the biogeographical knowledge of Trichoptera in the Balkan Peninsula. The collection data of caddisflies presented in this paper enhance our knowledge of distributional patterns for several species in South-Eastern Europe and Europe as a whole. Further investigations in Kosovo and other parts of Europe where caddisfly distributional data are scarce or lacking will enable better practical approaches in conservation plans and protection efforts for this group of aquatic insects.
